# Oxidative Stress-Induced Glomerular Mineralocorticoid Receptor Activation Limits the Benefit of Salt Reduction in Dahl Salt-Sensitive Rats

**DOI:** 10.1371/journal.pone.0041896

**Published:** 2012-07-24

**Authors:** Kento Kitada, Daisuke Nakano, Ya Liu, Yoshihide Fujisawa, Hirofumi Hitomi, Yuki Shibayama, Hirotaka Shibata, Yukiko Nagai, Hirohito Mori, Tsutomu Masaki, Hiroyuki Kobori, Akira Nishiyama

**Affiliations:** 1 Department of Pharmacology, Kagawa University, Kagawa, Japan; 2 Life Sciences Research Center, Kagawa University, Kagawa, Japan; 3 Department of Internal Medicine, School of Medicine, Keio University, Tokyo, Japan; 4 Department of Gastroenterology, Kagawa University, Kagawa, Japan; The University of Manchester, United Kingdom

## Abstract

**Background:**

Mineralocorticoid receptor (MR) antagonists attenuate renal injury in salt-sensitive hypertensive rats with low plasma aldosterone levels. We hypothesized that oxidative stress causes MR activation in high-salt-fed Dahl salt-sensitive rats. Furthermore, we determined if MR activation persisted and induced renal injury, even after switching from a high- to a normal-salt diet.

**Methods and Findings:**

High-salt feeding for 4 weeks increased dihydroethidium fluorescence (DHE, an oxidant production marker), p22phox (a NADPH oxidase subunit) and serum and glucocorticoid-regulated kinase-1 (SGK1, an MR transcript) in glomeruli, compared with normal-salt feeding, and these changes persisted 4 weeks after salt withdrawal. Tempol treatment (0.5 mmol/L) during high-salt feeding abolished the changes in DHE fluorescence, p22phox and SGK1. Dietary salt reduction after a 4-week high-salt diet decreased both blood pressure and proteinuria, but was associated with significantly higher proteinuria than in normal control rats at 4 weeks after salt reduction. Administration of tempol during high-salt feeding, or eplerenone, an MR antagonist (100 mg/kg/day), started after salt reduction, recovered proteinuria to normal levels at 4 weeks after salt reduction. Paraquat, a reactive oxygen species generator, enhanced MR transcriptional activity in cultured rat mesangial cells and mouse podocytes.

**Conclusions:**

These results suggest that oxidative stress plays an important role in glomerular MR activation in Dahl salt-sensitive rats. Persistent MR activation even after reducing salt intake could limit the beneficial effects of salt restriction.

## Introduction

Inappropriate regulation of the aldosterone/mineralocorticoid receptor (MR) system causes sodium retention and hypertension. Although MR antagonists are widely used for the treatment of chronic heart failure, studies have shown that they are also effective for the treatment of low-renin hypertension [1], [2], [3]. Interestingly, the anti-hypertensive effect of eplerenone, a selective MR antagonist, was not influenced by plasma aldosterone levels [2].

Increasing evidence has indicated that inappropriate MR activation contributes to the development of renal injury [4], [5], [6]. For example, the incidence of proteinuria was higher in patients with primary aldosteronism than in patients with essential hypertension [7], [8], and plasma aldosterone levels were positively correlated with urinary protein excretion in patients with chronic kidney diseases [9], [10]. However, MR antagonists have been shown to attenuate renal injury, especially glomerular injury, even under low circulating aldosterone levels, such as in salt-sensitive hypertension [11], [12], [13], [14]. We previously reported that high-salt feeding decreased plasma aldosterone levels, increased the expression of MR target-gene expression in microdissected glomeruli, and induced glomerular injury and proteinuria in Dahl salt-sensitive (DS) rats [11]. This evidence suggests that the contribution of MR to renal injury cannot be estimated simply by plasma aldosterone levels.

Recent studies have indicated that ligands and/or some pathological condition other than aldosterone induce MR activation and subsequent renal injury. We and others have demonstrated that glucocorticoids can contribute to the development of end organ damage through MR activation [15], [16], [17], [18]. High-glucose conditions augmented MR-dependent signaling in cultured rat mesangial cells (RMCs) [19]. Shibata et al. [20], [21] reported that Rac1, a member of the Rho family GTPases, worked as a potent activator of MR signal transduction and was related to salt-sensitive hypertension and renal injury. These findings suggest that not only aldosterone, but also other factors, could be involved in the activation of MR and subsequent renal injury under low plasma aldosterone conditions. However, the precise mechanisms by which renal MR is activated under relatively low aldosterone levels in salt-sensitive hypertension and renal injury has not been fully elucidated.

DS rats are widely used as a salt-sensitive hypertension model, and both high blood pressure [22] and renal oxidative stress [13], [23], [24], [25] have been implicated in the mechanism that leads to the development of renal injury in this model. However, the means by which oxidative stress contributes to the development of renal injury is incompletely understood. Tempol, a superoxide dismutase mimetic, has been used to examine the involvement of oxidative stress and renal injury in DS rats in several studies [12], [26], [27], [28]; however, the tempol dose used in these previous studies affected not only renal injury, but also blood pressure, and the possibility that the improvements in renal injury were caused indirectly by the blood-pressure-lowering effect could thus not be ruled out.

On the basis of these results, we hypothesized that oxidative stress-dependent MR activation contributes to renal injury in high-salt-fed DS rats, and we therefore investigated the effects of subpressor doses of tempol on the production of reactive oxygen species (ROS), glomerular MR activation, and proteinuria in DS rats. We also tested the hypothesis that MR activation could be sustained by high oxidant production even after reducing salt intake, which is currently the first choice treatment for salt-sensitive hypertensive patients. This could potentially induce rebound of the endogenous renin-angiotensin-aldosterone system (RAAS), substantially limiting the therapeutic effects of salt reduction because of synergism between the activated MR and increased aldosterone levels.

## Methods

### Animals and Experimental Protocols

Experimental protocols and animal care were performed according to the guidelines for the care and use of animals established by Kagawa University. The experiments were approved by the Animal Experimentation Ethics Committee at Kagawa University (No.57-1-2011). All experiments were performed using 6-week-old male DS rats (SLC, Shizuoka, Japan).

For the first series of experiments, rats were divided into three groups (n = 7 in each group): group 1, normal-salt diet (NS, 0.3% NaCl); group 2, high-salt diet (HS, 8% NaCl); and group 3, HS+tempol (0.5 mmol/L in drinking water). Preliminary experiments in the current study showed that tempol at this dosage had no effect on blood pressure, measured using a radiotelemetry system (TA11PA-C40 probes; Data Sciences International, St. Paul, MN) in high-salt-fed DS rats (mean arterial pressures at baseline and week 4, HS: 102±6 and 133±13 mmHg, HS+tempol: 102±4 and 139±5 mmHg, respectively, n = 3). The duration of the experiments was 4 weeks. In the second series of experiments, rats were divided into four groups (n = 10 in each group): group 1, NS (0–8 weeks); group 2, switched from HS (0–4 weeks) to NS (4–8 weeks) (HS-NS); group 3, switched from HS+tempol (0–4 weeks) to NS (4–8 weeks) (HS+tempol-NS); and group 4, switched from HS (0–4 weeks) to NS+eplerenone (100 mg/kg/day, orally; Selara, Pfizer, New York, NY) (4–8 weeks) (HS-NS+eplerenone). The experimental period was 8 weeks. Another group of rats (n = 4) underwent adrenalectomy (ADX) at week 4 of HS, and their diet was switched to NS for weeks 4–8. ADX was performed under anesthesia with sodium pentobarbital (50 mg/kg, intraperitoneally) as described previously [17]. Dexamethasone (20 µg/day, subcutaneously) was given as a glucocorticoid supplement in ADX rats [29].

We performed a further experiment to examine the efficacies of tempol and dietary salt reduction at different stages of renal injury. Rats were divided into three groups (n = 5): normal salt diet (0–12 weeks); switched from HS (0–8 weeks) to NS (8–12 weeks); and switched from HS+tempol (0–8 weeks) to NS (8–12 weeks). The experimental period was 12 weeks.

### Sample Collection

Systolic blood pressure (SBP) was measured during all experiments by tail-cuff plethysmography (BP-98A; Softron, Tokyo, Japan). Twenty-four-hour urine samples were collected once a week, starting after a 24-h acclimatization period in their metabolic cages. Rats were euthanized with an excessive dose of sodium pentobarbital (200 mg/kg). Arterial blood was collected from the abdominal aorta at the end of experimental period. The right kidney was snap-frozen in liquid nitrogen and stored at −80°C until processing for whole-kidney RNA extraction. Part of the left kidney was immersed in OCT compound (Sakura Finetek, Tokyo, Japan) and snap-frozen in chilled acetone for dihydroethidium (DHE) staining and glomerular RNA extraction.

### Laser-capture Microdissection, mRNA Isolation and Real-time Reverse-Transcription Polymerase Chain Reaction (RT-PCR)

For glomerular β-actin, serum and glucocorticoid-regulated kinase (SGK)1, Na+/H+ exchanger isoform (NHE)1 and p22phox mRNA analysis, glomeruli were microdissected using laser-capture microdissection methods [14], [17], [30]. Briefly, tissue in OCT compound was cryosectioned into 10-µm sections. Twenty-five glomeruli were randomly microdissected from each specimen under direct visualization with a laser-capture microscope (LM-200; Arcturus Bioscience, Mountain View, CA). Glomerular mRNA was extracted using RNAqueous Micro kits (Ambion, Austin, TX), according to the manufacturer’s protocol. Whole-kidney mRNA was extracted using the phenol-chloroform extraction method. mRNA levels in the glomeruli and whole kidney were analyzed by real-time RT-PCR using an ABI Prism 7000 with Power SYBR Green PCR Master Mix (Applied Biosystems, Foster City, CA). Primer information for SGK1, NHE1 and p22phox has been described previously [14], [17], [30]. The oligonucleotide primer sequences for rat β-actin were: sense: 5'-TCCACCCGCGAGTACAACCTT-3', antisense: 5'-ACGAGCGCAGCGATATCGTCAT-3'. All data are shown as relative differences after normalization to β-actin expression.

### DHE Staining in Kidney Sections

Frozen kidney segments in OCT compound were cut into 10-µm-thick sections and placed on a glass slide. DHE (10 µmol/L, Invitrogen, Carlsbad, CA) was topically applied to each tissue section [31]. Slides were incubated in a light-protected humidified chamber at 37°C for 30 min. Images were assessed using a laser scanning confocal microscope system (Bio-Rad Laboratories, Hercules, CA). DHE fluorescent images were visualized by excitation at 488 nm and emission at 610 nm to detect the oxidized DHE product ethidium. The average DHE fluorescence intensities of kidney cross sections (through cortex to inner medulla) and glomeruli were calculated from at least five sections (×100) or 10–20 glomeruli from each sample, respectively.

**Figure 1:**
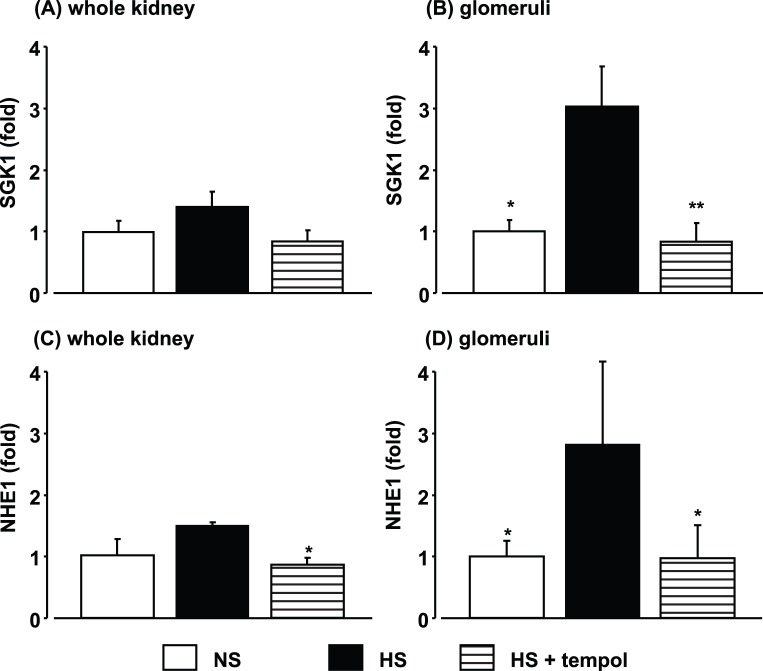
Effects of high-salt feeding and tempol treatment on expression of serum and glucocorticoid-regulated kinase (SGK)1 and Na^+^/H^+^ exchanger isoform (NHE)1 in whole kidney (A and C) and glomeruli (B and D) at 4 weeks after high-salt treatment. Data are expressed as means ± S.E.M.; n = 4 per group. *P<0.05, **P<0.01, compared to high-salt group.

**Figure 2:**
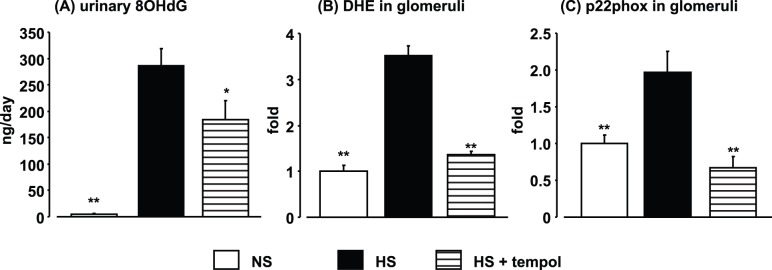
Effects of high-salt feeding and tempol treatment on oxidative stress markers evaluated by urinary 8-hydroxy-2'-deoxyguanosine (OHdG) excretion (A), dihydroethidium (DHE) staining in glomeruli (B), and p22phox mRNA expression in glomeruli (C) at 4 weeks after high-salt treatment. Data are expressed as means ± S.E.M.; n = 6 per group. *P<0.05, **P<0.01, compared to high-salt group.

### MR Luciferase Reporter Assay in Cultured RMCs and Mouse Podocytes

RMCs and conditionally-immortalized mouse podocytes were used and maintained as previously reported [19], [32], [33]. MR transfection of RMCs (passage 5–7) and mouse podocytes was performed using the Lipofectamine (Invitrogen) transfection method [34]. At 24 h after subculturing, cells were subjected to transient transfection with the MR luciferase reporter for 24 h [34]. After transfection, the cells underwent serum-free culture for 24 h and were then treated with aldosterone (1 nmol/L) and the ROS generator paraquat (100 µmol/L), with or without eplerenone (10 nmol/L) or tempol (100 µmol/L) for 24 h. For the luciferase assay, 40-µL cell extracts were used and the assay was carried out according to the manufacturer’s instructions (ToyoInk, Tokyo, Japan).

**Figure 3:**
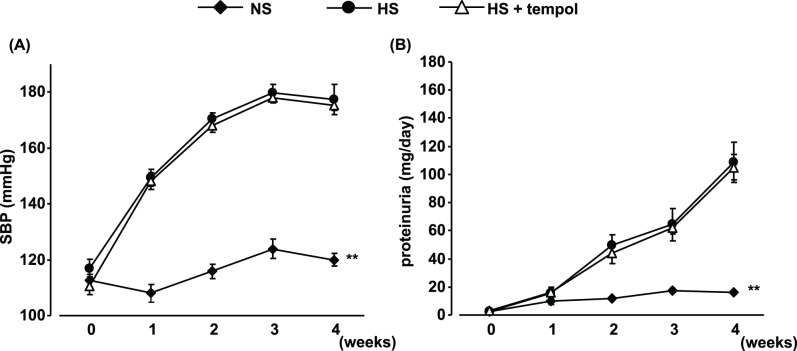
High-salt-feeding-induced changes in systolic blood pressure (SBP) (A) and proteinuria (B) and effects of tempol treatment. Data are expressed as means ± S.E.M.; n = 7 per group. **P<0.01, compared to high-salt group.

### Other Analytical Procedures

Urinary 8-hydroxy-2'-deoxyguanosine (8-OHdG) and protein excretion were determined using commercially available kits (Nikken Seil, Shizuoka, Japan, and microTP-test, Wako, Osaka, Japan, respectively). Plasma aldosterone concentrations were also analyzed using a commercially available kit (SPACK-S aldosterone kit; TFB, Tokyo, Japan).

**Figure 4:**
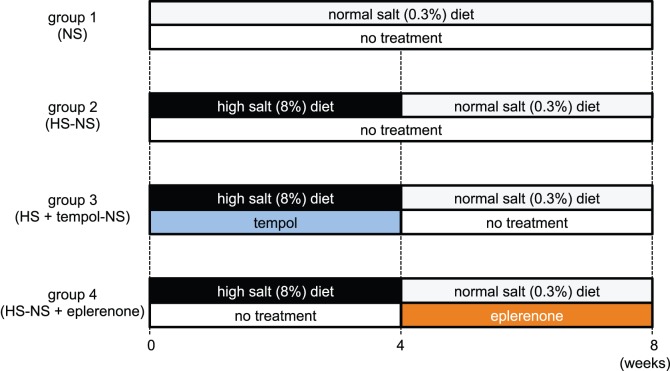
Rats were divided into four groups (n = 10 in each group): group 1, NS (0–8 weeks); group 2, switched from HS (0–4 weeks) to NS (4–8 weeks) (HS-NS); group 3, switched from HS+tempol (0–4 weeks) to NS (at 4–8 weeks) (HS+tempol-NS); and group 4, switched from HS (0–4 weeks) to NS+eplerenone (100 mg/kg/day, orally, 4–8 weeks) (HS-NS+eplerenone). Tempol was withdrawn at week 4 in group 3. Eplerenone treatment was started from week 4. The experimental period was 8 weeks.

### Statistical Analysis

All values are expressed as the mean ± S.E.M. Relevant data were processed using InStat (Graph-PAD Software for Science, San Diego, CA). Statistical analysis was performed using one-way analysis of variance followed by Dunnett’s multiple comparison tests. Differences were considered significant at *P*<0.05.

**Table 1:** Time-dependent changes in plasma aldosterone levels (pg/mL) before and after salt intake, and effects of tempol.

	Week 0	Week 4	Week 5	Week 8
	HS (and tempol)		NS	
HS-NS	139.3±22.8	98.8±15.9	293.8±39.4	295.0±54.3
HS+tempol-NS	157.3±45.2	57.3±18.1	215.0±35.7	327.5±46.4

Data are expressed as means ± S.E.M.; n = 4 per group. HS: high salt, NS: normal salt.

## Results

### SGK1 and NHE1 Expression and Oxidative Stress in 4-week High-salt-fed DS Rats

Four-week high-salt feeding did not increase the levels of SGK1 and NHE1 mRNAs, transcriptional targets of MR [35], [36], [37], in whole kidney samples (Figure 1A and 1C), whereas significant increases in SGK1 and NHE1 mRNA levels were observed in laser-captured glomeruli from HS rats (Figure 1B and 1D). The increases in glomerular SGK1 and NHE1 expression were markedly suppressed in the HS+tempol group, indicating that MR signaling was augmented in glomeruli in high-salt-fed DS rats, as reported previously [11]. We also reported previously that the increase in glomerular SGK1 expression in this model was prevented by eplerenone [11]. Renal oxidative stress was analyzed using three different methods. Urinary 8-OHdG excretion was markedly increased in HS rats after 4 weeks of high-salt feeding (Figure 2A). DHE fluorescence was also increased in both low-power-field kidney cross sections (2.0±0.1 fold vs. 1.0±0.2 fold in NS rats, *P*<0.01) and glomeruli (Figure 2B) in HS rats after 4 weeks of high-salt feeding. In addition, a high-salt diet increased mRNA expression of p22phox, a subunit of NADPH oxidase, in laser-captured glomeruli in HS rats (Figure 2C). Tempol treatment suppressed the increases in all three markers of oxidative stress (DHE fluorescence in low power field; 1.3±0.1 fold, *P*<0.01) in HS rats.

**Figure 5:**
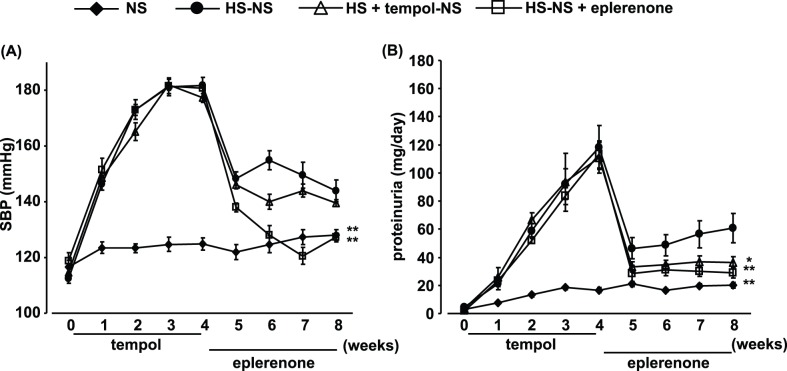
Time-dependent changes in systolic blood pressure (SBP) (A) and proteinuria (B) before and after reducing salt intake, and effects of tempol (0–4 weeks; HS + tempol-NS) and eplerenone (4–8 weeks; HS-HS + eplerenone) treatment. Data are expressed as means ± S.E.M.; n = 10 per group. *P<0.05, **P<0.01, compared to high salt (HS)-normal salt (NS) group.

### Blood Pressure and Proteinuria

High-salt feeding time-dependently increased SBP and proteinuria (Figure 3). Tempol treatment (0.5 mmol/L in drinking water) did not affect the increases in SBP and proteinuria in 4-week high-salt-fed DS rats.

### Analysis of Effects of Salt Reduction

The protocol for this experiment is described in Figure 4. After 4 weeks of high-salt feeding in DS rats, high salt-fed animals that were not treated with tempol were separated into two groups: rats that received dietary-salt reduction by feeding a normal-salt diet for an additional 4 weeks (HS-NS: group 2 in Figure 4); and rats that received both eplerenone treatment and dietary-salt reduction for an additional 4 weeks (HS: group 4 in Figure 4). In addition, rats treated with tempol for the first 4 weeks of the high-salt diet were switched to no tempol and dietary-salt reduction for an additional 4 weeks (HS+tempol-NS: group 3 in Figure 4). Rats that received a normal-salt diet for 8 weeks were used as a normal control (NS: group 1 in Figure 4).

**Figure 6:**
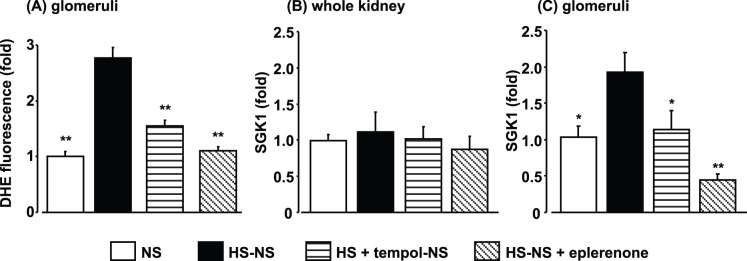
Dihydroethidium (DHE) staining in glomeruli (A), and serum and glucocorticoid-regulated kinase (SGK)1 expression in whole kidney (B) and glomeruli (C) at 8 weeks, and effects of tempol (0–4 weeks) and eplerenone (4–8 weeks) treatments. Data are expressed as means ± S.E.M.; n = 6 per group. *P<0.05, **P<0.01, compared to high salt (HS)-normal salt (NS) group.

### Plasma Aldosterone Levels and Effects of Salt Reduction

Plasma aldosterone levels were increased after reducing the salt intake, and the increased levels were maintained until the end of the experimental period. There was no significant difference in plasma aldosterone levels between the tempol-pretreated and untreated groups (Table 1).

### Effects of Salt Reduction on Blood Pressure in High-salt-fed DS Rats

SBP was decreased in the HS-NS group after reducing the salt intake (Figure 5A), but was still slightly higher than that in the NS group at week 8 (4 weeks after salt reduction). Tempol pretreatment had no effect on these changes in SBP, except the value at 1 week after salt reduction. Meanwhile, eplerenone treatment, which was started with salt reduction, normalized SBP in DS rats.

### Effects of Salt Reduction on Proteinuria in High-salt-fed DS Rats

Proteinuria was clearly reduced in the HS-NS group after reducing the salt intake (Figure 5B); however, even after salt reduction, levels still remained significantly higher than in the NS group. Tempol treatment only during high-salt feeding further suppressed proteinuria, to a significantly lower level than in the HS-NS group. Eplerenone treatment started with salt reduction strongly suppressed proteinuria to the level seen in tempol-pretreated animals.

**Figure 7:**
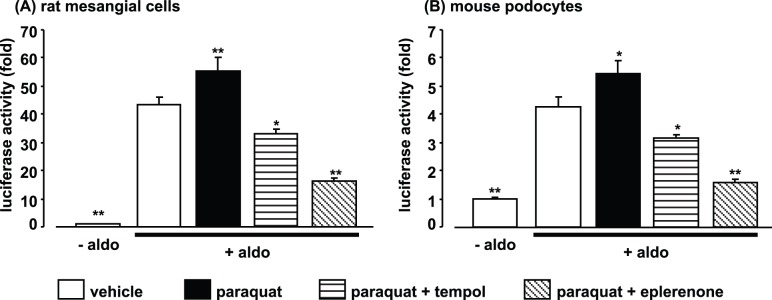
Effects of paraquat and eplerenone or tempol on mineralocorticoid receptor luciferase reporter assay in cultured rat mesangial cells (A) and mouse podocytes (B). Data are expressed as means ± S.E.M.; n = 6 per group. *P<0.05, **P<0.01 compared to aldosterone+vehicle group. aldo: aldosterone.

Tempol treatment only during high-salt feeding induced similar results in animals with more severe proteinuria after an 8-week high-salt diet (proteinuria at 4 weeks after salt reduction: NS; 20.9±3.3 mg/day, HS-NS; 126.9±23.1 mg/day, HS+tempol-NS; 78.3±19.2 mg/day, *P*<0.05).

### Effects of Salt Reduction on Oxidative Stress and MR Activation in Kidney of DS Rats

DHE fluorescence was greater in kidneys in the HS-NS group compared with the NS group, even at 4 weeks after salt reduction (Figure 6A). Tempol or eplerenone treatment attenuated DHE fluorescence in the kidneys in DS rats.

At 4 weeks after reducing salt intake, there were no significant differences in SGK1 expression in whole kidneys among the groups (Figure 6B). SGK1 expression in the glomeruli, however, was significantly higher in the HS-NS group than in the NS group (Figure 6C), even at 4 weeks after reducing the salt intake. Tempol pretreatment and eplerenone treatment with salt reduction both attenuated glomerular SGK1 expression in HS-NS rats. The increased glomerular NHE1 expression at week 4 in HS rats was no longer observed at week 4 after salt reduction (NS: 1.00±0.12 fold, HS-NS: 0.96±0.16 fold).

### Effects of ADX on Blood Pressure and Proteinuria after Salt Reduction

An increase in plasma aldosterone could inhibit normalization of proteinuria after salt reduction. In order to eliminate the effects of a ‘rebound’ increase in plasma aldosterone, rats were subjected to ADX at week 4 of HS. Reducing salt intake in these animals lowered blood pressure, and there was no difference in SBP between animals with or without adrenal glands at week 4 after salt reduction (144±4 vs. 141±8 mmHg, respectively). In contrast, urinary protein excretion in animals with intact adrenal glands (61±10 mg/day) was significantly attenuated at week 4 after ADX (35±3 mg/day, *P*<0.01).

### MR Luciferase Reporter Assay in Cultured RMCs and Mouse Podocytes

To further examine the role of ROS in MR transcriptional activity, we investigated the effect of the ROS generator paraquat on the aldosterone-induced increase in MR luciferase activity in RMCs and mouse podocytes. Paraquat significantly enhanced MR reporter activity (Figure 7A), and the increase was almost completely suppressed by tempol in RMCs. In addition, eplerenone exhibited a stronger inhibitory effect on MR reporter activity than tempol. Similar results were observed in mouse podocytes (Figure 7B).

## Discussion

MR-dependent signaling has been reported to be augmented in the kidneys in salt-sensitive hypertensive animals, despite their relatively low plasma aldosterone levels, and MR antagonists have demonstrated reno-protective effects, even at subpressor dosages [11], [12], [13]. There are several possible explanations for this dissociation between the efficacy of MR antagonists and plasma aldosterone levels. The possible inverse agonist characteristics of MR antagonists could be responsible [38]; however, even if MR antagonists reduced MR activity to a lower level than that in normal healthy controls, this cannot explain the enhanced MR-dependent signaling in the kidney of salt-sensitive animals. We and others recently reported that several factors, such as cytosolic Rac1 [20], [21] and glucocorticoids [17], play roles in MR activation in vivo, independent of plasma aldosterone levels. In the present study, concomitant increases in renal ROS production and glomerular MR activation in DS rats were produced by 4 weeks of high-salt feeding, and these changes were suppressed by tempol, suggesting that high-salt-feeding-induced oxidative stress caused MR activation in the glomeruli of DS rats. The current in vitro luciferase assays also confirmed that oxidative stress can accelerate MR transcriptional activity in the cells that comprise the glomeruli. This may explain why MR is activated in salt-sensitive hypertension. However, the detailed mechanism whereby oxidative stress activates MR is unclear. Rac1 is a component of NADPH oxidase, and the increase in oxidative stress and MR activity could thus be partially a consequence of Rac1 activation. A previous study, however, demonstrated that renal injury induced by Rac1-mediated MR activation was not attenuated by tempol in vivo [21]. It therefore seems likely that oxidative stress mediates MR activity through mechanisms other than the Rac1-dependent pathway. Further studies to evaluate the relationships between oxidative stress and conformation, location, and epigenetic changes of MR are needed to clarify this issue.

Proteinuria is an important marker for the development of chronic renal injury [39], [40]. Several studies have shown that excessive ROS production and/or renal MR activation plays an important role in the development of hypertension, proteinuria and renal injury in salt-sensitive hypertensive animals [11], [12], [13]. We therefore expected that the suppression of ROS production and MR activity by tempol would also suppress proteinuria during weeks 0–4 in the current experiment. However, there was no significant difference in proteinuria between tempol-treated and untreated HS rats, indicating that the activation of glomerular MR by oxidative stress under the current experimental conditions may not play a predominant role in the development of proteinuria in DS rats. We previously demonstrated that 3 mmol/L tempol, which was 6-fold the dose used in the current study, did suppress the blood pressure increase and proteinuria and normalize the increase in thiobarbituric acid-reactive substances in high-salt-fed DS rats [41]. The results of the current study showed that a subpressor dose of tempol (0.5 mmol/L) only induced a partial reduction of urinary 8-OHdG excretion, a marker of systemic oxidative stress, but abolished the increase in glomerular ROS production markers, suggesting that tempol at this dose somehow scavenged ROS in glomeruli more effectively than in other parts of the body. Regarding the involvement of MR in blood pressure, it is possible that extra-glomerular MR activities, such as in the distal nephron [20] and brain [42], [43], may be more important, and we failed to observe any significant increases in SGK1 and NHE1 expression levels in whole kidney samples. The reason why high-salt feeding only increased MR activity in the glomeruli thus remains unclear.

Restricted dietary-sodium intake has been widely recommended for treating hypertension [44]. Meanwhile, reducing salt intake is also known to stimulate endogenous RAAS [45], [46], [47], [48]. In the present study, blood pressure and proteinuria in DS rats were decreased after switching from a high- to a normal-salt diet, accompanied by an increase in plasma aldosterone levels. The risk induced by this increase in RAAS might have been underestimated in the therapy for hypertension, because the benefit induced by salt reduction is apparent. However, the present study showed that reducing salt intake resulted in limited changes in blood pressure and proteinuria, and that additionally suppressing MR activity, either during high-salt intake (by tempol) or during salt reduction (by eplerenone or ADX) elicited further improvements in proteinuria. Furthermore, glomerular MR activity, which was increased by oxidative stress, remained at high levels even after reducing salt intake. In addition, tempol and ADX showed no additional suppression of blood pressure after salt reduction, while eplerenone further reduced SBP to normal levels. These results suggest that increased MR activity, together with increased plasma aldosterone after salt reduction, could synergistically limit the beneficial effects of dietary-salt reduction on proteinuria in DS rats, and that these effects appear to be independent of the effect on blood pressure after dietary-salt reduction.

Eplerenone strongly attenuated proteinuria in DS rats in the present study. Similar protective effects have been reported for candesartan, an angiotensin II type 1 receptor antagonist [49], and atorvastatin, a 3-hydroxy-3-methylglutaryl-coenzyme A inhibitor [50], in DS rats with sustained oxidative stress even after dietary-salt reduction. These results are consistent with the current study. The anti-oxidative effects of candesartan or atorvastatin are implicated in their protective mechanisms [49], [50], suggesting the possibility that they may induce their protective effects via the same pathway as in the present study. It is also possible that candesartan prevented the effects of an increase in RAAS after salt reduction, including the increase in plasma aldosterone, though the changes in plasma aldosterone and MR activity were unfortunately not measured in these studies.

SGK1 transcription is reported to respond to the other stimuli, such as glucocorticoid receptor stimulation [51]. However, we previously demonstrated that eplerenone markedly suppressed the increase in SGK1 expression in the glomeruli of high-salt-diet-fed DS rats [11], indicating that a high-salt diet induced an MR-dependent response in DS rats. We also observed that the sustained increase in SGK1 after dietary-salt reduction was abolished by eplerenone in the present study. In addition, NHE1 expression, another transcript target of MR, was increased in the glomeruli of DS rats after a 4-week HS diet, though this increase had disappeared at week 4 after dietary-salt reduction. Taken together, these results suggest that high-salt feeding stimulated glomerular MR in DS rats.

Recently, Morizane et al. [52] reported the time course of changes in adrenal aldosterone synthesis in DS rats. They demonstrated that initial 8% high-salt feeding (at weeks 2 and 5) decreased adrenal aldosterone synthesis, which was well correlated with plasma aldosterone level and is consistent with the results of the current study (at week 4). However, surprisingly, high-salt feeding increased both adrenal aldosterone synthesis and plasma aldosterone at week 7 in DS rats, compared to normal-salt feeding, and the phenomenon was not observed in Dahl salt-resistant rats. The biphasic response of plasma aldosterone suggests that there are ‘early’ (up to 5 weeks) and ‘late’ (after 7 weeks) stages in the involvement of aldosterone/MR in the pathophysiology of DS rats. The present study demonstrated that 8% salt feeding induced MR activation within 4 weeks, suggesting that oxidative stress-induced MR activation already occurred at the ‘early’ stage. We also found that tempol could enhance the anti-proteinuric effect of dietary-salt reduction after 8 weeks of high-salt feeding (‘late’ stage), indicating the importance of oxidative stress during high-salt feeding at both stages.

Reducing salt intake increases plasma aldosterone levels [45], [46], [47], [48]. However, the role of increased plasma aldosterone levels in the development of tissue damage is unclear. In an animal study, Mori et al. showed that increased plasma aldosterone levels induced by a low-salt diet promoted myocardial fibrosis via MR in volume-overloaded rats [53]. Several clinical studies have claimed that reducing salt intake showed no effect or increased the risk of cardiovascular events [54], [55], [56]; however, significant controversy exists regarding this issue [57], [58], [59]. The findings of the current study may shed light on this difficult problem. The conditions when dietary-salt restriction is started (oxidative stress-induced MR activation in the current study) could influence the therapeutic outcome of salt restriction. Treatment with MR blockers in addition to salt restriction may thus prove a more effective therapy for preventing and inhibiting the development of renal injury in salt-sensitive hypertension.
